# Total skin electron beam therapy rationalization and utility of *in* *vivo* dosimetry in a high-volume centre

**DOI:** 10.1259/bjro.20190008

**Published:** 2019-07-20

**Authors:** Sarah Misson-Yates, Marium Naeem, Isabel Palmer, Eleanor Holden, Owen Hedley, Mark McGovern, Stephen Morris, Anthony G Greener

**Affiliations:** 1 Department of Medical Physics, Guy’s and St Thomas’ NHS Foundation Trust, SE1 9RT, London, UK; 2 Department of Clinical Oncology, Guy’s and St Thomas’ NHS Foundation Trust, SE1 9RT, London, UK

## Abstract

**Objective:**

This paper reports on the rationalization of a substantial pool of *in vivo* dosimetry (IVD) data from patients treated with total skin electron beam therapy (TSEBT) and the application of this to verify the accurate delivery of TSEBT when changing linac manufacturer.

**Methods:**

Thermoluminescent dosimeter IVD data from 149 patients were analyzed comparing the population mean and standard deviation for each site. The number of sites required to confirm the prescribed dose were reviewed considering both dosimetric and clinical relevance. The reduced sites were then used to assess the continued dosimetric accuracy on new equipment and the results were compared statistically using the Mann–Witney test.

**Results:**

The trunk dose measurement points were reduced from nine to six and five extra trunk sites were identified and reviewed clinically prior to removal.

Following change in manufacturer the trunk dose points showed no statistically significant change and confirmed that patients had received within 1.3% of the intended mean trunk dose using both delivery methods.

A statistically significant change in 4 out of the 13 extra trunk sites was seen following the move to the new centre. However, all but one site showed a change of less than 1 standard deviation.

**Conclusion:**

The total number of measurement points per patient were reduced from 27 to 19 which constituted a 25% saving in preparation and read out.

Accurate delivery of prescribed dose was confirmed following measurement point reduction for treatments delivered on linacs from two different manufacturers.

**Advances in knowledge:**

Proven methodology for rationalization of IVD measurements for TSEBT

## Background and purpose

Mycosis fungoides is the most common form of cutaneous T-cell lymphoma;^1^ however, it is still uncommon at a population level, with an incidence in England of 0.75 per 100,000.^2^ A number of treatments have been used historically, including pharmacotherapy, ultraviolet therapy and radiotherapy.^1^ Early radiotherapeutic treatments used superficial X-rays which are still used today in conjunction with total skin electron beam therapy (TSEBT) which has been used since the 1950s.^3^ Recently, alternatives have been employed such as helical tomotherapy.^4^

TSEBT has undergone a degree of evolution and there is intercentre variation in delivery. The most common technique is the dual-beam Stanford technique,^5^ with some variations such as treating with the patient prone/supine^6^ or introducing a rotational beam component.^7^ *In vivo* dosimetry (IVD) is used in TSEBT treatment for three purposes: to confirm correct delivery of prescribed dose to the trunk, to monitor extremity doses to avoid under- or overdose due to patient positioning errors (and in some centres to determine boost doses), and to monitor organ at risk doses, specifically the lens of the eye. A systematic literature review of the use of IVD for TSEBT treatments was recently performed by Guidi et al^8^. The paper summarized the range of data published to date and the different types of IVD used.^8^ The most widely used IVD method for TSEBT treatments are thermoluminescent dosimeters (TLDs) and semiconductor diodes. The largest studies cited by Guidi et al^8^ were Antolak et al,^9^ using TLDs for 72 patients with 22 readings which infers 1584 measurements in total and Yaparpavi et al^10^ using diodes, for 360 patients which resulted in 809 measurements. The use of TLDs have also been validated through their use in external TSEBT dosimetry audits.^11^ Other methods such as Radio-chromic film, Optically Stimulated Luminescence Devices and Metal Oxide Semiconductor Field Effect Transistors have been assessed for TSEBT, however the studies have been limited by low patient numbers.^8^

From 2006 to 2018, more than 200 TSEBT patients have been treated at our centre. This has resulted in over 4800 measurements providing a substantial datapool for analysis in comparison to the studies reported by Guidi et al.

The service has changed in that time from being delivered on Elekta Precise (Elekta, Crawley, UK) units between 2008 and 2016 to Varian TrueBeams^©^ (Varian, California, USA) from 2017; although the modified Stanford Technique has continued to be used and standard fractionation regimens maintained. The standard fractionation regimes used are; 30 Gy in 20 fractions over 5 weeks, 24 Gy in 16 fractions over 4 weeks or 12 Gy in 8 fractions over 2 weeks.

IVD is performed for the first fraction of each TSEBT course using LiF:Mg,Ti TLD-100. The average of multiple trunk dose points confirm delivery of the prescription dose and the extra trunk locations confirm adequate dose coverage, and that any shielding used is sufficient.^12^ Due to the large data set, accrued experience and workload associated with the high number of TLDs per patient the number and positions of measurement points were reviewed. Following implementation of the TLD site reduction, the revised sites were then used to assess the accuracy of treatment delivery and compare *in vivo* doses between the Elekta Precise and Varian TrueBeam delivery methods

The aim of this paper is to report on, the review and reduction of measurement sites, based on their standard deviation and clinical importance, of a substantial pool of *in vivo* dosimetry data from mycosis fungoides (MF) patients treated with TSEBT. The application of this approach is then used to verify the accurate delivery of TSEBT when changing linac manufacturer.

## Method

From 2006 to 2016, treatments took place using an Elekta Precise Linac; the patients were positioned at 350 cm source to surface distance, on a custom-built stand, with a 3.4 mm thick Perspex screen placed in front to degrade the electron beam from a nominal beam energy of 6 MeV to approximately 4.2 MeV at the patient surface (mean energy at the surface estimated from the single beam percentage depth ionization measurements). The patients were treated with six dual high dose rate (3000 cGy/min) electron fields, each fraction, with a 35° hinge angle.

Patients have Perspex foot and leg shielding (12 or 24 mm) for the anterior and posterior treatment positions. The hands are shielded superiorly at some of the treatment positions in Perspex hand shields. The use of shielding is customized per patient based on disease extent and to reduce toxicity to uninvolved areas. Lead covered goggles are also used to minimize the lens doses throughout the whole treatment.

IVD was performed at the first fraction for all TSEBT patients. This utilized 27 measurement points, 18 extra trunk and nine trunk locations; at each point a sachet containing two lithium fluoride "TLD-100" chips were attached to the patients’ skin.

For each fraction a batch calibration was performed; five TLDs were irradiated to a known dose and a calibration factor, corrected for machine output, was calculated. Each TLD chip had an individual calibration factor, and readings were corrected for supralinearity. After irradiation, the chips were read out using a Harshaw 5500 TLD reader. Due to the 50 chip capacity of the TLD reader, the chips had to be read out in two batches, a process which could take up to an hour. The results were then entered into a database, where they were compared with an aggregate mean and standard deviation of previous patients’ measurements. Unexplained results deviating by ±1 standard deviation (SD) were repeated for that patient and reviewed again. The overall uncertainty of measured dose at each point is estimated to be around 3.5% (1 SD) taking into account batch calibration and individual chip correction factors and utilizing two TLD chips per sachet. This precision is within the standard deviation seen for individual sites from TSEBT treatments which range from around 5 to 30%.

**Figure 1.**
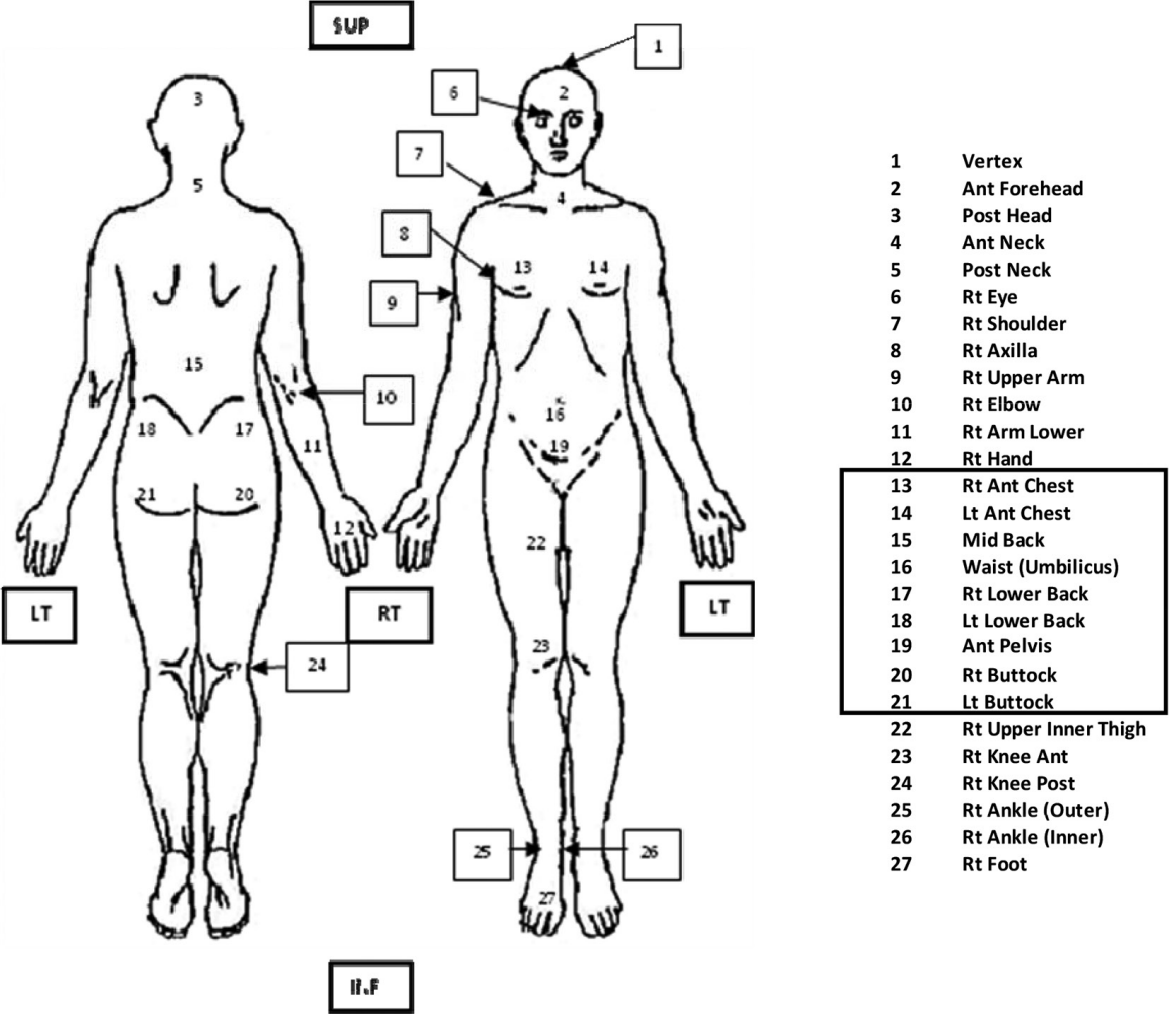
Clinical TLD positions with trunk dose measurement points indicated. TLD, thermoluminescent dosemeter.

Data from the first 149 patients (treated on the Elekta Precise) were analyzed, with the nine trunk dose measurements points (Figure 1, points 13–21) considered separately from the extra trunk points. For the trunk dose measurements, the mean dose received, and the standard deviation of each point were compared. The number of points required to confirm the prescribed dose was reviewed and any points of greater uncertainty were identified for possible removal taking into account both dosimetric and clinical relevance.

For the extra trunk dose measurement points, comparison was made based on the interpatient standard deviation at each point, and the overall variation. The mean and standard deviation of the lens dose measurements across all the patients were also reviewed based on the EORTC recommendation that the dose to the lenses should be less than 15% of the prescribed dose.^12^

In January 2017, the TSEBT service had to be moved to a Varian TrueBeam due to the opening of a new Cancer Treatment Centre. Therefore, the technique was recommissioned with the key aim to ensure the techniques were dosimetrically comparable, using the same treatment stand. On the TrueBeam, the isocentre height is higher and the nominal isocentric field size for high dose rate electron treatments is smaller (36 × 36 cm compared to 40 × 40 cm on the Elekta Precise). Therefore, in order to achieve comparable dosimetry and sufficient vertical uniformity across the field, the SSD had to be increased from 350 to 400 cm SSD, and the hinge angle adjusted from 35.0° to 34.6°.

The IVD data from the first 18 patients treated on the Varian machines was compared to the Elekta data to review the dosimetric equivalence of the two machines. The statistics package IBM SPSS Statistics was used to test the data sets for normality using the Shapiro–Wilk test, and compare the data sets from the two treatment units using the Mann–Witney test.

## Results

### Trunk dose data

The principle aim of the trunk dose measurements is to verify that the prescription dose has been delivered correctly. In TSEBT, treatments the dose is often prescribed to the waist/umbilicus point however this relies on the accuracy of a single data point. Due to the uncertainty in measured dose from a single point the TSEBT technique in our centre is prescribed to the mean trunk dose.

Table 1 summarizes the mean and SD of the doses measured at each trunk measurement location with the mean doses normalized to the prescription dose per fraction of 150 cGy. The mean dose delivered to the trunk using all nine measurement locations was 98.6% of the prescription dose (mean SD of points 9.7%) which verifies that on average the patients received within 1.4% of the prescribed dose per fraction.

**Table 1.** Summary of trunk dose measurement points

**Number**	**TLD position**	**Normalized to prescription**	
		**Mean** (**% of prescribed dose**) (***N* = 149, Elekta pre reduction**)	**SD** **(% of prescribed dose)** **(*N* = 149, Elekta pre reduction)**
**13**	Rt Ant chest	100.1	7.6
**14**	Lt Ant chest	99.8	11.3
**15**	Mid back	98.8	9.8
**16**	Waist	96.3	9.9
**17**	Rt lower back	100.8	8.6
**18**	Lt lower back	101.0	6.5
**19**	Ant pelvis	92.2	15.7
**20**	Rt buttock	99.9	7.6
**21**	Lt buttock	98.7	10.3
**Mean dose nine positions**		98.6	9.7
**Mean dose five positions**		100.1	8.8
**Mean dose six position post reduction of sites (*N* = 40)**		98.9	8.6

SD, standard deviation; TLD, thermoluminescent dosimeter.

The Ant pelvis measurement point had the largest standard deviations (15.7%) compared to the other sites, most likely due to inconsistencies in positioning the TLD and self-shielding, and so was deemed to be an unstable site and removed from use. The three other sites, the waist and both buttock positions, were deemed to provide duplicate information to other sites (*e.g.* Lt buttock was positioned very close to Lt lower back) and thus these were also considered for removal.

On reviewing the measurement locations, a move to just five trunk measurement points was considered to provide sufficient information to assess the mean trunk dose. The retention of the LT and RT points (chest and back) allows for assessment of any ‘leaning’ of the patient anteriorly or posteriorly. For these five measurement positions, the mean trunk dose was found to be 100.1% of the prescription dose (mean SD of points 8.8%) which was in close agreement to the mean for all nine original trunk–dose sites. However, these points do not include an anterior inferior measurement point which is required to verify that all the fields have been delivered. Therefore, the introduction of an additional trunk point was considered necessary and a "Rt Ant pelvis" measurement point, located on or directly above the anterior ilium, was added giving a total of six trunk measurements.

### Extra trunk dose data

In contrast to the trunk measurement points, extra trunk locations have no bearing on determining the successful delivery of the prescription dose; they provide dose information for mobile, potentially self-shielded, or intentionally shielded locations. Shielded sites such as the hands and feet should be monitored with TLDs to detect if these areas are under dosed, and it is of particular importance to assess lens dose as lead goggles are used throughout treatment to shield the eyes and reduce the risk of cataract formation.

Table 2 summarizes the results for the 18 extra trunk measurement points with the mean doses normalized to the prescription dose per fraction of 150 cGy. Any points with an SD greater than 20% were thought to be potentially unstable and are shown in light grey and any sites which are intentionally shielded during treatment are shaded in dark grey. These sites were discussed with the clinical lead so that clinically relevant measurement points were not removed.

**Table 2.** Summary of results for all 18 extra trunk measurement sites

**Measurement location**	**Number**	**Mean (% of prescribed dose)**	**SD (% of prescribed dose)**
**Vertex**	**1**	99.3	34.2
**Ant forehead**	**2**	85.3	21.4
**Post head**	**3**	107	24.6
**Rt axilla**	**8**	72.8	22.5
**Rt upper inner thigh**	**22**	57.9	29.5
**Ant neck**	**4**	98.6	8.1
**Post neck**	**5**	101.2	15.3
**Rt shoulder**	**7**	92.8	17.9
**Rt upper arm**	**9**	100.9	6.8
**Rt elbow**	**10**	109.1	16.7
**Rt arm lower**	**11**	91.6	11.3
**Rt knee ant**	**23**	98.8	9.2
**Rt knee post**	**24**	101.7	9.2
**Rt eye**	**6**	**5.5**	**3.2**
**Rt hand** *also shielded	**12**	**84.5**	**7.7**
**Rt ankle outer**	**25**	**76.9**	**13.3**
**Rt ankle inner**	**26**	**80.8**	**12.1**
**Rt foot**	**27**	**99.7**	**14.6**
**Mean extra trunk dose**	**All sites**	86.9	15.4
	**Excl. shielded areas**	93.6	17.4
**Mean extra trunk dose (5 sites removed) pre reduction**	**Excl. shielded areas**	89.2	17
				**Mean extra trunk dose post-reduction of sites (*N* = 40)**	**Excl. shielded areas**	87.3	17.9

SD, standard deviation.

Dark Grey: Shielded areas

Light Grey: SD>20%

Overall the mean extra trunk dose (unshielded) is 93.6% SD 17.4% and the highest SD for an unshielded location is 29.5% for the RT Upper Inner Thigh measurement. These data compare well to published values Anacak et al^13^ found the mean extra trunk dose deviated by 19.7% (SD17.7%) for 58 patients. Weaver et al^14^ demonstrated SD ranging from 5 to 24% for dose to tangential surfaces with the maximum variability seen for inner thigh measurements at 24%.

Based on the data in Table 2, measurement points providing duplicate positional information were removed, particularly where a high SD suggested greater variation in population data, *e.g.* "Post Neck" or "Rt Elbow." Even where the SD was high, dose measurement points under intentional shielding were universally retained, *i.e.* eyes, hands, and feet. These principles led to the removal of the "Vertex" (1), "Post Neck" (5), "Rt Shoulder" (7), "Rt Axilla" (8), "Rt Upper Arm" (9), "Rt Elbow" (10) and "Rt Upper Inner Thigh" (22). Following clinical input, the "Rt Axilla" and "Rt Upper Inner Thigh" were retained as these were deemed to be clinically useful sites for considering toxicity, despite their high standard deviation due to variable self-shielding, leaving a total of 13 extra trunk measurement points.

It can be seen from Table 2 that the mean eye dose was 5.5%, however in some cases this was found to be as high as 14.9%. TLD measurements for patients with high measured doses such as this were repeated with advice given to the treatment radiographers to ensure the goggles were securely strapped on the patients’ head. The measurements confirm that the eye shielding was sufficient to keep the dose below the EORTC recommendations of 15% of the prescribed dose.

The final number of sites proposed was 6 trunk dose measurement points and 13 extra trunk dose points. Including the five calibration chips, this constitutes a 25% reduction in the number of TLDs needing to be prepared and analyzed per patient and, coincidentally, reduces the total number of TLD chips per patient to below the 50 chip capacity of the Harshaw 5500 reader, significantly reducing the read procedure time.

### TLD data following the move to varian TrueBeam linacs

The reduced sites method was used to monitor 40 patients on the Elekta Precise linacs prior to moving to the Varian TrueBeam’s. The mean trunk and extra trunk results post-reduction of sites are given in Tables 1 and 2. The mean trunk dose post-reduction verified that on average patients were receiving within 1.1% of the prescribed dose with a SD of 8.6% which agrees well with 1.4 and 9.7% pre-reduction. The mean extra trunk dose also showed good agreement pre- and post-reduction. The mean extra trunk dose (unshielded) was 87.3% with a SD of 17.9% post-reduction compared to 89.2% (SD 17.0%) pre-reduction.

Overall the data showed good agreement and the reduced sites IVD method was used to assess the IVD for the Varian TrueBeam patients.

**Figure 2.**
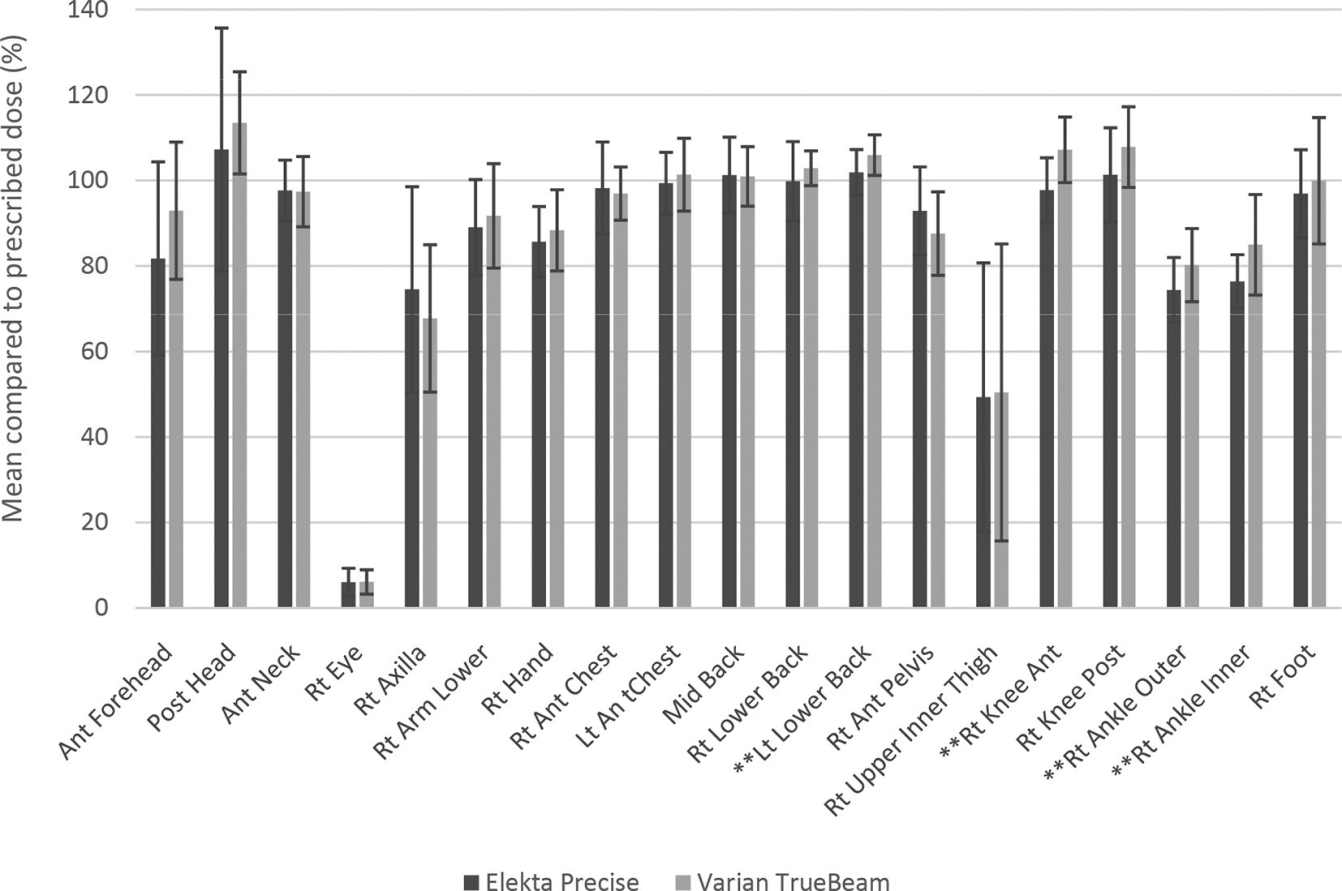
Comparison of TLD results for patients treated on the Varian TrueBeam and Elekta Precise Linacs. **Statistically significant changes were seen for LT Lower Back, RT Knee Ant, RT Ankle Inner and RT Ankle Outer. TLD, thermoluminescent dosemeter.

The IVD results for the first 18 patients treated on the Varian TrueBeam were collated and are shown in Table 3. The results were compared to the data from the Elekta Precise treatments (post-TLD site reduction, *n* = 40), and are displayed in Figure 2. Most sites received a comparable dose to those received from the Elekta Precise treatments. All but one site ("Rt Knee Ant") showed a change of less than 1 SD.

**Table 3.** Measurement results for the first 18 patients treated on the Varian TrueBeam

**Measurement location**	**Number**	**Elekta mean** **(% of prescribed dose)**	**Varian mean** **(% of prescribed dose)**	**Elekta SD** **(% of prescribed dose)**	**Varian SD** **(% of prescribed dose)**	**Change from Elekta Precise** **(%)**
**Ant forehead**	**1**	81.8	92.9	22.6	16.1	**13.7**
**Post head**	**2**	107.2	113.5	28.4	11.9	**5.8**
**Ant neck**	**3**	97.6	97.4	7.1	8.2	−**0.3**
**Rt eye**	**4**	6.1	6.1	3.3	2.8	**1.2**
**Rt axilla**	**5**	74.5	67.8	24.0	17.2	−**9.1**
**Rt arm lower**	**6**	89.1	91.7	11.1	12.2	**3.0**
**Rt hand**	**7**	85.7	88.4	8.3	9.5	**3.2**
**Rt Ant chest**	**8**	98.2	96.9	10.8	6.2	−**1.3**
**Lt Ant chest**	**9**	99.4	101.4	7.2	8.5	**2.0**
**Mid back**	**10**	101.2	101.0	8.9	7.0	−**0.3**
**Rt lower back**	**11**	99.9	102.9	9.2	4.1	**3.0**
**Lt lower back**	**12**	101.9	105.9	5.3	4.7	4.0*
**Rt Ant pelvis**	**13**	92.9	87.6	10.3	9.8	−**5.7**
**Rt upper inner thigh**	**14**	49.3	50.5	31.4	34.7	**2.3**
**Rt knee ant**	**15**	97.7	107.2	7.6	7.7	9.7*
**Rt knee post**	**16**	101.3	107.8	11.0	9.4	**6.4**
**Rt ankle outer**	**17**	74.4	80.2	7.6	8.6	7.8*
**Rt ankle inner**	**18**	76.4	85.0	6.3	11.8	11.3*
**Rt foot**	**19**	96.9	100.0	10.3	14.8	**3.1**

SD, standard deviation.

* p<0.05 statistically significant change

The Shapiro–Wilk test found that all but three of the 19 sites’ data ("Rt Arm Lower", "Mid Back" and "Rt Knee Post") deviated significantly from a normal distribution on at least one of the two treatment units (*p* > 0.05). Therefore, a non-parametric test was used to assess the change in measured doses upon the move to the new centre.

Using the Mann–Witney test, it was found that the measured doses to four sites had increased significantly (*p* < 0.05) following the move to the Varian TrueBeam: "Lt Lower Back" (*U* = 531, *p* = 0.004), "Rt Knee Ant" (*U* = 584, *p* = 0), "Rt Ankle Outer" (*U* = 482, *p* = 0.024) and "Rt Ankle Inner" (*U* = 0.001). These are indicated in Table 3. All other sites showed no statistically significant change.

The "Rt Eye" was still well below the EORTC recommendation that the dose to the lenses should be less than 15% of the prescribed dose.^12^

Table 4 summarizes the mean dose and SD for the trunk measurement points after the reduction in TLD sites, and after the move to the new treatment centre. The table also includes trunk data from recent papers.^13,15^

**Table 4.** Mean and standard deviations for Elekta Precise and Varian TrueBeam measurement points

	**Mean (% of prescribed dose)**	**Mean SD (% of prescribed dose) of sites**
**Elekta** - Trunk (*N* = 40)	98.7	8.6
**Varian data -** Trunk (*N* = 18)	99.3	6.7
**Anacak et al^13^**	Not stated	7.4
**Elsayad et al^15^**	Not stated	<10

SD, standard deviation.

## Discussion

The review of the initial IVD data led to a reduction in the number of trunk dose TLD measurements points from 9 to 6, and the extra trunk sites from 18 to 13 this constituted a 25% time saving in TLD preparation and read out for physics staff. The extra trunk SD values compared well with published values.^13,14^

After the change in treatment delivery from Elekta Precise to Varian TrueBeam the population mean trunk dose increased by 0.9% from 148.0 ± 6.1 cGy to 149.3 ± 4.3 cGy (Table 4). This is a small difference and the trunk dose points showed no statistically significant change. Therefore, on average patients received within 1.3% of the prescribed dose to the trunk using either delivery method which confirms accurate delivery using both types of treatment machine.

The value of IVD was demonstrated when a first fraction overdose of 23% was identified when treating the first clinical case on the Varian TrueBeam. Under national guidelines, this dose was classed as much greater than intended and was reported to the appropriate external body. An external audit had taken place prior to commencing treatments. The root cause analysis found that the commissioning and external audit had taken place without the treatment stand and the additional scatter from this and the interpatient variability in trunk dose had contributed to the overdose. The recommendation was that a "test dose" should have been performed following commissioning to allow for such variabilities. This discrepancy was immediately corrected and the mean trunk dose verified following repeat measurement on the second fraction.

There is variation between patients and the population mean trunk dose SD was 8.6 and 6.7% for Elekta and Varian deliveries respectively. This compares well with published data, Anacak et al^13^ 7.4% and Elsayad et al^15^ <10%. Across the extra trunk measurement points only 4 of 13 sites, LT Lower back, RT Knee Ant, RT Ankle inner and outer showed a significant dosimetric change (Table 3).

**Figure 3.**
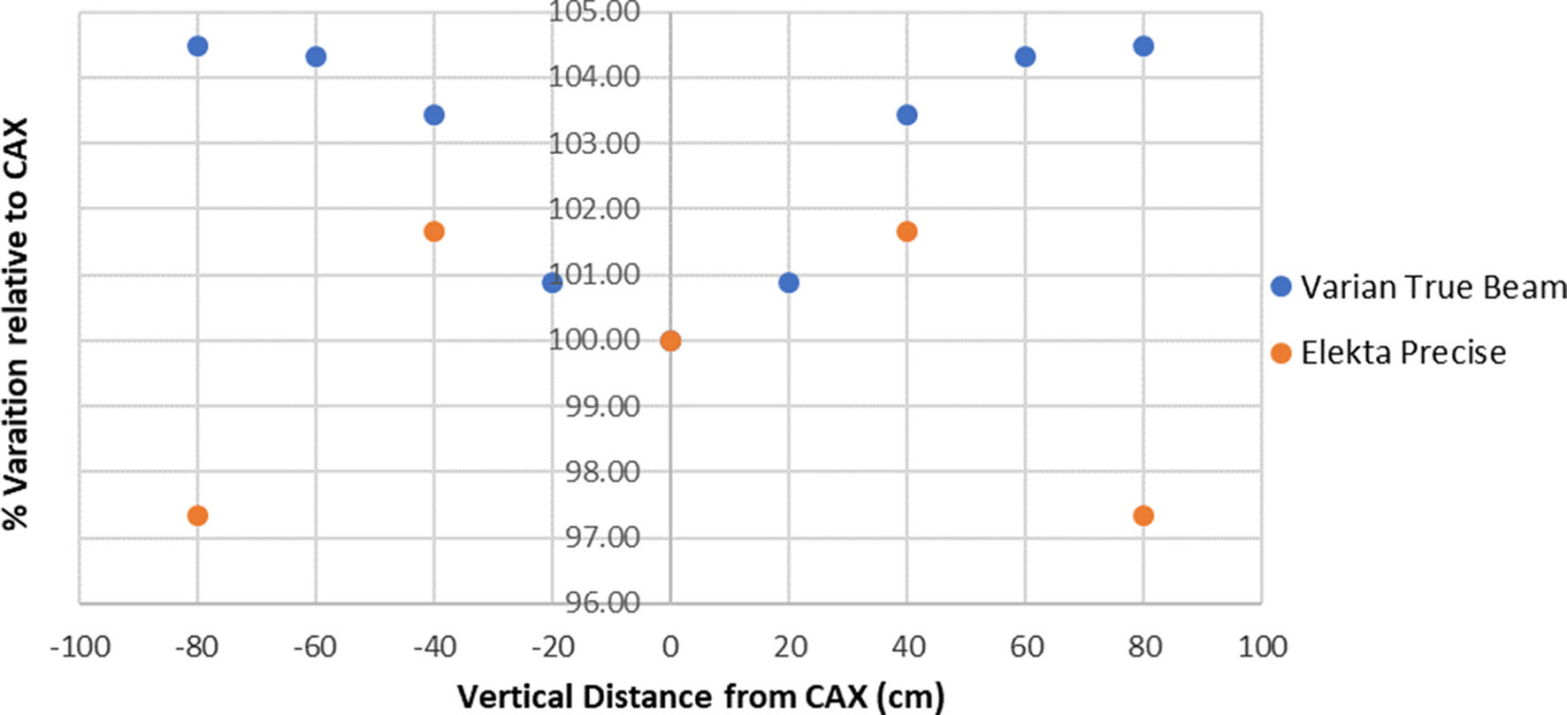
Vertical field homogeneity at the CAX for Elekta Precise *vs* Varian TrueBeam.

At commissioning, the field homogeneity of the beam was measured to check compliance with the EORTC criteria. The EORTC states that the beam dose homogeneity in air should be within 10% across the treatment dimensions and within 8% vertically and 4% horizontally within 160 x 60 cm.^1^ Commissioning measurements were performed in air at the patient plane using Scanditronix–Wellhofer EDP-5-3G diodes attached to a wooden rod suspended from the ceiling. In Figure 3, the central axis profiles are shown in the vertical direction for the Elekta Precise and Varian TrueBeam linacs. The data are limited for the Elekta Precise linacs due to the difficulty of measuring, in air, at the patient plane.

The data clearly show that both linacs are within 8% along the vertical central axis (CAX) within ±80 cm and that the Varian TrueBeam has a significantly higher flatness compared to the Elekta Precise of +7.1%. This is reflected in the Varian TrueBeam doses below knee which are consistently higher (on average +7.7%) than the Elekta doses in the same region. This explains the statistically significant differences seen in the knee and ankle doses. Whilst the increase in dose to the ANT forehead is not statistically significant, it could potentially be attributed to the change in flatness depending on the patients’ height. However, the patient’s height is not routinely recorded so it is not possible to analyze the data to look for any direct correlation. The increase in dose at the knees and ankles has not resulted in changes to shielding since moving the technique to the Varian TrueBeam.

The key issue in selecting dose measurement points for TSEBT is separating out the interpatient and intrafraction errors. An ideal measurement point in IVD is one that has a small intrafraction variation and is of clinical relevance. This ensures a single measurement taken on a given fraction can be scaled up to derive an accurate estimate of total treatment dose at that point. For whole body treatments such as total body photon irradiation and TSEBT, there are numerous measurement locations which will exhibit a range of intra fractional variations for individual patients as well as interpatient variation for the same measurement locations. The ideal way to assess this would be to take repeated measurements across multiple fractions for a representative group of patients. Measurement points with small interfractional variation but large interpatient differences are ideal in showing up genuine differences in skin doses received between patients.

## Conclusions

This paper describes the rationalization and optimization of TSEBT IVD measurement points and the transfer of this methodology to confirm accurate delivery using equipment from two different linac manufacturers.

IVD forms an essential part of TSEBT treatment delivery and TLDs provide an effective means of assessing patient dosimetry provided the sites are well chosen and reviewed regularly.
